# Study protocol for FIBROKIT: a new tool for fibromyalgia diagnosis and patient follow-up

**DOI:** 10.3389/fneur.2023.1286539

**Published:** 2023-11-21

**Authors:** Laura Lucena del Amo, Elena Durán-González, Jorge A. Ramírez-Tejero, Antonio Martínez-Lara, David Cotán

**Affiliations:** Pronacera Therapeutics S.L., Seville, Spain

**Keywords:** fibromyalgia, gut microbiota, oxidative stress, proteomics, mitochondria, metagenomics

## Abstract

Fibromyalgia (FM) is a complex disease that is characterized by chronic musculoskeletal pain and has great economic impact. FM prevalence is about 2% to 4% worldwide, affecting mainly middle-aged women, and its complex pathophysiology complicates diagnosis, treatment and the findings of solid biomarkers. Previous studies have suggested an association between the disease and oxidative stress, mitochondrial metabolism, intestinal microbiota and inflammation, providing sufficient data to support the multifactorial origin of FM. Hence, the objective of this randomized, prospective, low-interventional, double-blinded and placebo-controlled clinical trial is the development of a specific panel of FM biomarkers and the evaluation of their response to a six-month nutritional intervention based on the Mediterranean diet supplemented with extra virgin olive oil (EVOO). For this purpose, the experimental design implies the recruitment of a large cohort of female Spanish patients. Middle-aged women who meet the diagnostic criteria for FM according to the American College of Rheumatology (ACR) will be eligible, along with age-matched healthy women. Both groups will be randomly divided into placebo (olive oil, OO) and treatment groups (extra virgin olive oil, EVOO), and will provide samples at the beginning (T_0_), after 3 months of nutritional intervention (T_1_), at the end of the nutritional intervention in 6 months (T_2_), and 6 months after the end of nutritional intervention (T_F_), being enrolled for 1 year. Data will be collected through health questionnaires, and whole blood and stool samples will be taken and analyzed. Blood will be used for western-blotting and proteomic analysis of mitochondrial homeostasis and plasma proteome, while stool will undergo metagenomic analysis, respectively. This study represents the first low-interventional investigation with more than 200 participants focused on exploring the association of oxidative stress, mitochondrial metabolism, intestinal microbiota and related pathways with a nutritional intervention in the context of FM. As a result, the outcomes of this study will significantly contribute to the development of a comprehensive and robust panel of diagnostic biomarkers, and will shed some light on their modulation with non-pharmacological therapies such as nutrition.

**Clinical trial registration**: https://clinicaltrials.gov/study/NCT05921409, identifier: NCT05921409.

## 1 Introduction

Fibromyalgia (FM) is a limiting chronic syndrome characterized by widespread musculoskeletal pain accompanied by a wide variety of related symptoms such as fatigue, sleep disturbances and other cognitive disturbances. FM prevalence is estimated to range from 2% to 4% (1), primarily affecting adult women from developed economies, although men may be underdiagnosed (2). Currently, diagnosis is mainly based on the criteria of the American College of Rheumatology (ACR), which include the presence of widespread pain affecting both sides of the body and occurring above and below the waist. Traditionally, exploration of tender points was also used for diagnosis, establishing the threshold at a minimum of 11 out of 18 pain points, although this diagnostic procedure is becoming outdated. In fact, these criteria were revised in 2016 to require widespread pain in at least 11 out of 11 defined regions (not just points) of the body (1). In any event, many patients still go undiagnosed due to several factors and suffer from a marked decrease in their quality of life and work productivity, which leads to significant economic impact on society (3). Pathophysiology of FM remains elusive, leading to a complete absence of trusted biomarkers for its diagnosis and treatment. FM is a complex condition which includes several symptoms affecting both physical and psychological aspects. From the physical point of view, FM is frequently accompanied by long-lasting fatigue, pain, joint stiffness, dry eyes and mouth and several genitourinary problems. In psychological terms, anxiety, depression and very low self-esteem stand out (4).

Traditionally, FM has been associated with oxidative stress and mitochondrial dysfunction. Oxidative stress is defined as an imbalance between reactive oxygen species (ROS) and antioxidant levels in the body. The production of ROS appears to be primarily attributed to the mitochondria, which are unique energy-producing organelles in cells and have their own genome. Excess ROS or compromised mitochondrial antioxidant defense result in detrimental effects on essential biomolecules such as DNA, proteins, and lipids (5). This impairment can result in compromised mitochondrial function (6). Moreover, it triggers the liberation of pro-apoptotic proteins from the mitochondrial intermembrane space, initiating the process of cell death activation, which is known as mitophagy (7). This process governs the removal of damaged mitochondria, whereas the opposite process, called mitochondrial biogenesis, generates new functional organelles (7). Some authors have conducted studies to investigate variance in mitochondrial mass protein levels among chronic fatigue syndrome (CFS) and FM patients (8). The studies have revealed that FM patients exhibit lower levels of the peroxisome proliferator-activated receptor-gamma coactivator 1 alpha (PGC-1α) and the mitochondrial transcription factor A (TFAM), whereas CFS patients did not display significant changes in these proteins. TFAM plays a crucial role in human mitochondrial DNA transcription and works as a key regulator of mitochondrial DNA copy numbers. These discrepancies in mitochondrial protein levels between the two conditions offer valuable insights into their respective pathogenesis. Moreover, they hold the potential to facilitate the development of novel biomarkers that can effectively differentiate between CFS and FM. Recent research has demonstrated a reduction in the activity of antioxidant enzymes such as superoxide dismutase 1 (SOD1), catalase (CAT) and nicotinamide adenine dinucleotide phosphate (NADPH), which correlates with the severity of pain and fatigue in FM patients (9). Furthermore, Altindag and Celik (10) observed lower total antioxidant capacity (TAC) among individuals with FM, and multiple studies have shown in FM patients decreased levels of CoQ10, a relevant enzyme for mitochondrial function (7). Additionally, mitochondrial imbalance in FM manifests itself as an increased expression of autophagic genes and the elimination of damaged mitochondria (7). Mitochondria-derived ROS play a key role in stimulating the activation of mediator signaling molecules, including the nuclear transcription factor kappa-B (NF-κβ). Consequently, this activation leads to an up-regulation of inflammatory cytokines, such as interleukin-1β (IL-1β) and tumor necrosis factor-α (TNF-α), and FM has consistently been linked to a low-grade inflammation profile. Notably, NF-κβ is activated by a critical type of membrane receptor associated with oxidative stress and inflammation. The regulation of this receptor serves as the mechanism through which certain antioxidant molecules can effectively inhibit the inflammatory process (11). The significant impact of ROS and inflammation extends across a wide range of unrelated diseases, including nervous system disorders, gastrointestinal autoimmune conditions and even FM. There is substantial evidence indicating a connection between mitochondrial dysfunction and elevated inflammation levels in FM (9, 11), with patients exhibiting higher plasma levels of interleukin-6 (IL-6) and interleukin-8 (IL-8) compared to healthy individuals (12). In fact, there is evidence suggesting that the levels of circulating cytokines in FM patients play a role in its pathophysiology (13).

Recently, several authors have undertaken studies to identify biomarkers linked to FM by analyzing plasma proteomic profiles using advanced proteomic techniques. Ramírez-Tejero et al. (14) identified 33 proteins exhibiting differential expression in FM patients compared to controls, with a majority of proteins associated with inflammatory processes. Another study (15) revealed differences in proteins involved in inflammatory, metabolic, and immune processes, demonstrating a correlation between these proteins, patients’ pain perception and psychological distress. Han et al. (16) discovered 22 differentially expressed proteins in FM patients compared to pain-free controls primarily associated with blood coagulation processes, immune response and interactions with extracellular matrix receptors. Collectively, these findings highlight the role of coagulation, inflammation and immune response in the pathogenesis of the disease.

The direct relationship of mitochondrial functioning with energy consumption in certain human tissues makes the musculoskeletal and nervous systems more dependent on ATP metabolism and, consequently, on mitochondrial homeostasis. Insofar as muscles, the degenerative alterations observed in muscle membranes, mitochondria and capillary vessels in FM could potentially be linked to deficiencies in membrane ion channeling, oxygen and metabolite transport, as well as ATP production through oxidative phosphorylation (17). In the context of the nervous system, recent studies have described FM as a disorder involving pain regulation and central sensitization (1), as well as a nociplastic disease (18), as pain management is among the most affected factors of the pathology, whose patients usually suffer from hyperalgesia and allodynia. The enhancement of secondary hyperalgesia is proposed to be driven by the activation of N-methyl-D-aspartate (NMDA) receptors in the spinal cord’s dorsal horn and periaqueductal gray region, leading to central sensitization. Neurons containing NMDA receptors become hypersensitive to stimulation when their ATP levels decrease, resulting in increased NMDA activity, temporal summation of pain and long-term potentiation and causing prolonged synaptic activity (12). In fact, it has been proved that individuals with FM exhibit alterations in pain regulation, such as increased neuronal activity in regions associated with pain processing (1), higher levels of excitatory neurotransmitters such as glutamate and lower levels of inhibitory neurotransmitters such as serotonin and norepinephrine (18). Furthermore, control of behavior and cognitive functioning is among the primary functions of the central nervous system (CNS). Studies have shown that alterations in the levels of antioxidant enzymes are linked to the underlying mechanisms of specific psychiatric disorders, including depression and anxiety, which are two of the most prevalent symptoms in FM (17).

It is widely recognized that the CNS plays a part in the development of FM, although the precise mechanism behind this connection is still under investigation. However, the gut-brain axis has become an interesting field to explore. Gut microbiome refers to the complete collection of bacteria, archaea, fungi, viruses, protozoa and helminths that exist in the gastrointestinal tract, as well as their genome and different metabolites. Throughout adulthood, the gut microbiota of a healthy individual is primarily dominated by four main phyla: *Bacteroidia*, *Firmicutes*, *Actinobacteria* and *Verrucomicrobia*. However, as individuals progress into old age, the microbial composition of the gut undergoes notable changes. Specifically, there is an increased proportion of *Bacteroides* spp. observed, along with distinct abundance patterns of *Clostridium* groups that differ between elderly individuals and younger adults (19). Its composition is dynamic and is influenced by both the host and the surrounding environment (20). A significant breakthrough has been made in understanding how the microorganisms residing in our gut can actively regulate brain function and behavior. This remarkable connection implies various components like the CNS, the enteric nervous system (ENS) and the sympathetic and parasympathetic branches of the autonomic nervous system, as well as neuroendocrine and neuroimmune pathways (21). This bidirectional communication between the gut and the brain enables the CNS to exert its influence over critical functions of the gastrointestinal (GI) tract. Remarkably, it also allows signals originating from the GI tract to have an impact on CNS function in return (19). The gut-brain axis plays a vital role in maintaining homeostasis and regulating a wide range of physiological functions. It encompasses motor, sensory, autonomic and secretory functions of the GI tract, influencing processes from energy metabolism to mood regulation. This axis relies on an intricate network of communication and involves extensive neuronal connections with afferent fibers projecting from peripheral tissues to higher-order processing centers in cortical CNS structures, as well as efferent projections from the CNS to the smooth muscle in the intestinal wall (22). The signaling within this axis operates through various interconnected mechanisms which include the activation of afferent sensory neurons of the vagus nerve, neuro-immune pathways and neuroendocrine pathways, as well as the involvement of microbial metabolites such as short-chain fatty acids (SCFAs) and microbial-derived neurotransmitters (21). Remarkably, some bacteria exhibit receptors for certain neurotransmitters and can even produce substances that allow them to communicate at the CNS level. Through their interaction with the primary role of the vagus nerve, these bacteria can modulate the activity of the hypothalamic-pituitary-adrenal (HPA) axis, showcasing the profound impact of the gut-brain axis on health homeostasis (23). Some bacterial neurotransmitters are noradrenaline, acetylcholine, GABA and serotonin. While serotonin is predominantly recognized for its role as a neurotransmitter in the brain, an astonishing 95% of serotonin in the body is actually found within the gut (22). This neurotransmitter plays crucial roles in regulating mood, cognitive functioning and central processing of sensory signals involved in pain processes (24). Stress is believed to be a contributing factor to alterations in the integrity of the epithelial barrier in the gut. This compromised barrier can lead to increased intestinal permeability, allowing gram-negative bacteria to translocate across the mucosal lining. Consequently, this situation enables direct interactions between humoral and cellular mediators with immune cells and the ENS, triggering an immune response characterized by heightened production of inflammatory mediators (22). As a result, various studies have observed changes in intestinal microbiota composition in several disorders, including gastrointestinal, rheumatic and metabolic conditions (20). Strikingly, Clos-García et al. (25) discovered a decrease in some bacterial strains in FM patients, specifically those associated with a healthy microbiome. These strains are involved in the production of SCFA and the depletion of *Firmicutes* phylum abundance. Additionally, the study revealed that the most affected metabolic pathways were connected to neurotransmitters such as glutamate and nitric oxide, highlighting once again the central role of the CNS in this syndrome.

The dietary habits of individuals play a key role in influencing the gut microbial community and its metabolic activities. Among healthy dietary choices, Mediterranean-style diets stand out, as they are associated with the prevention of various diseases, including cardiovascular and metabolic disorders, colorectal cancers and other chronic and acute processes (26). The positive effects of such diets are likely due to their high content of fibers, mono-and polyunsaturated fatty acids, antioxidants and polyphenols (26). Research has shown that adopting a Mediterranean-style diet leads to improvements in serum inflammation biomarkers and gene expression profiles (nutrigenomics) (27). Olive oil is an essential element in the Mediterranean diet and is known for its beneficial properties. Olive oil polyphenols possess a range of health benefits, including antioxidant, anti-inflammatory, antimicrobial, antiviral, anti-atherogenic, anti-thrombotic, anti-mutagenic and hypoglycemic characteristics. With regard to oxidative stress, consumption of high-phenol olive oil can help reduce cellular oxidative damage. Furthermore, it has the potential to lower the concentration of inflammatory markers and to enhance antioxidant capacity (28). Testing of this nutritional intervention as a therapeutic treatment in FM patients has shown that beneficial results are obtained in relation to oxidative DNA damage and antioxidant levels (29). These results could be explained by the close relationship between nutrient absorption and an adequate mitochondrial function.

In summary, FM is a chronic and complex condition that is highly underdiagnosed due to the lack of reliable biomarkers that might facilitate accurate diagnosis and disease monitoring. Hence, the primary focus of this study is to develop a targeted panel of specific plasma and fecal biomarkers capable of diagnosing this condition from readily obtained biological samples. Furthermore, the secondary objective is to investigate how nutrition can influence the modulation of these biomarkers. Identification of accurate biomarkers through this study is the key to advancing our understanding and management of FM, ultimately leading to improved patient outcomes.

## 2 Materials and methods

### 2.1 Study design

**Figure 1:**
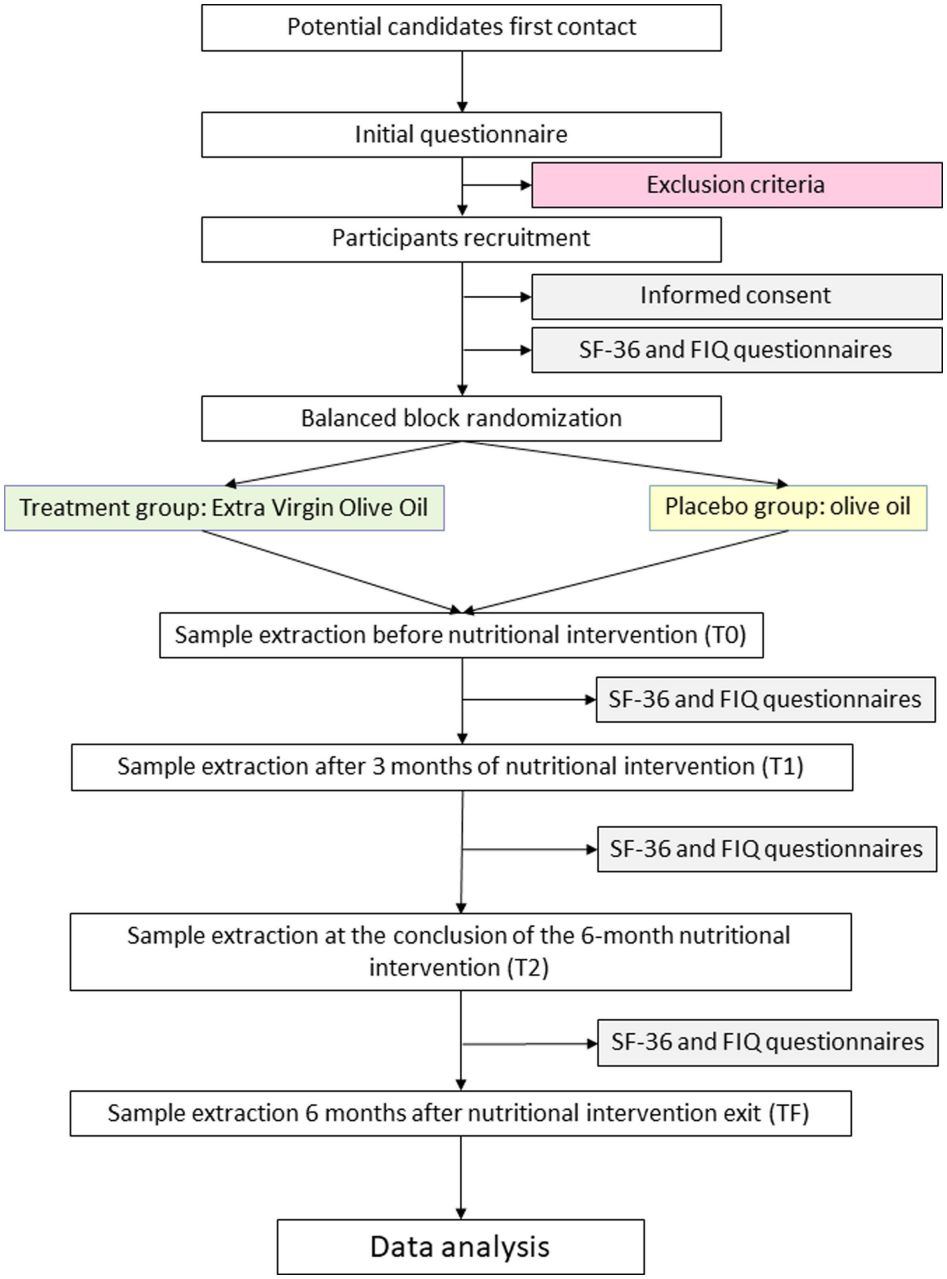
Flow diagram of this study.

This is a randomized, prospective, low-interventional, double-blinded and placebo-controlled clinical trial. This study will be performed on adult women who meet ACR diagnostic criteria for FM (FM group), along with age-matched healthy volunteers (CONTROL group). The double-blinded, placebo-controlled, randomized experiment will be conducted by using extra virgin olive oil (EVOO) as treatment and olive oil (OO) as placebo, being under nutritional intervention in adherence to a Mediterranean diet supplemented with one of the olive oils for 6 months since the first sample collection. The participants will be assigned to each group randomly, using the balanced block randomization method. Briefly, once participants have been chosen for project enrollment, they will be tagged with an ordinal numeric code with three numbers by an employee from the company not related with the project nor the laboratory. This same employee will decide the codification of treatment (EVOO) and placebo (OO) groups, by assigning them an “A” or a “B” letter. Then, a second employee neither related with project or lab workflow, will randomly allocate these codes to 8-individuals blocks composed by 4 patients and 4 healthy volunteers. These blocks will be assigned to a treatment group by following the pattern ABBAABBA. In this process, this second employee will only have access to the date and time in which the participation form was completed as a unique identifier, as well as the condition of the participant: patient (FM) or healthy volunteer (CONTROL). This process will ensure that no one can link any code to its health condition, neither to participants’ data. Both processes will be reflected in patients’ database and documented in a password encrypted word file that will be also kept physically in a key-protected drawer during the entire project. The blinding method will be maintained until the data have been fully analyzed, and once this information is required to write the scientific communications that will be generated. As a result, both, patients and researchers will be fully blinded about treatment to be taken and health conditions from each participant code. Furthermore, by following the balanced block randomization method, the treatment and placebo group will comprise the same number of patients and healthy individuals. The study will span a total duration of 12 months, during which four blood and stool samples will be collected from each participant: the first before nutritional intervention (T_0_), the second after 3 months of nutritional intervention (T_1_), the third at the conclusion of the six-month nutritional intervention (T_2_) and the fourth 6 months after the end of the intervention (T_F_). This final timepoint have been included to check whether the identified biomarkers that response to nutritional intervention would change back again to the baseline, indicating a strong dependance between nutritional habits and these specific biomarkers. Figure 1 illustrates the workflow that will be followed in this study.

### 2.2 Participants and recruitment

#### 2.2.1 Sample size calculation

According to the most detailed research conducted in Spain, the estimated prevalence of FM among the population is 2.4%. Specifically, among women of 40 to 49 years of age, the prevalence is 8.4%, whereas for women between 50 and 59, it stands at 6.7% (30). With this data, we will set the expected prevalence in 7.5% (mean value between 8.4% and 6.7%) and calculate sample size with the *Z*-level of confidence (1.96), the expected prevalence *p* (7.5) and the precision (*d*, calculated as *P*/2) the following formula:

$$n=\frac{Z^{2}P\left(1-P\right)}{d^{2}}$$

With this calculation, a 95% confidence interval (CI) with an α level of 0.05 and an additional 10% based on an estimation of patients’ withdrawal, a minimum of 206 participants is required. The statistical power will be set at 0.80 (*β* = 0.2).

#### 2.2.2 Inclusion criteria

A total of 250 participants will be recruited for the study through an online survey launched across FM associations and social media in order to effectively reach a larger pool of potential participants. To be eligible, the participants must meet the following inclusion criteria:

- Age range of 40 to 59 years, since these women are the most affected population group (30).
- Maintenance of a sedentary lifestyle and adherence to a balanced diet following the principles of the Mediterranean diet, in order to limit the observed changes to those related with nutritional habits
- Confirmed FM diagnosis by a healthcare professional according to 1990 ACR criteria.

Exclusion criteria for the study are as follows:

- Individuals outside the age range of 40 to 59 years.
- Participants who have engaged in structured or planned physical activity in the last month, such as medium or high intensity directed classes or scheduled aerobic/anaerobic exercise for more than 2 days a week.
- Individuals who are either underweight (BMI <18.5) or severely obese (BMI >35.9).
- Those diagnosed with any form of cancer, cardiovascular disease (e.g., atherosclerosis, cardiomyopathy), autoimmune diseases (e.g., lupus, celiac disease, thyroiditis) or metabolic disorders (e.g., diabetes, metabolic syndrome).
- Participants who have undergone pharmacological treatment with non-steroidal anti-inflammatory drugs, analgesics, antidepressants or antioxidants in the month preceding the study.
- Consumption of alcohol exceeding 12 g/day, as specified in the Mediterranean diet.

Before the beginning of the project, participants will complete an informed consent form and a patients’ information datasheet (Supplementary file S1) that have been approved by the Ethical Committee for Research with Medications (CEIM) at the Quirónsalud-Catalunya Hospital Group (protocol code IDI-20210749, approval record No. 01/2022).

#### 2.2.3 Adherence

To ensure adherence, each participant will be given individualized attention. The assigned nutritionists will maintain regular contact with the participants to assess their progress with the intervention, following a contact plan based on two phone calls per month. In addition, a phone line and a specific mail inbox (fibromialgia@pronacera.com) will be created for queries, complaints and management of sample collection appointments. All the participants experiencing adverse event such as gastrointestinal complications, constipation, acute diarrhea or severe weight loss or gain will be immediately evaluated by nutritionists and proposed for withdrawal if those effects are somehow related to the nutritional intervention. Besides, the phone line will be uses for instant messaging communication with patients, to send them reminders about clinical appointments, solve some doubts about menus and olive oil consumption or adverse events assistance. A proactive communication plan has been designed to collect information regarding the reasons for the participants’ missed appointments, inactivity, or withdrawal. Additionally, in case of withdrawal or sample collection missing, the data collected will be used in the analysis, although upcoming datapoints will be unavoidably missed. Even though we will analyze a fully completed group of 250 participants at starting point (T_0_), the groups are expected to decrease while the project goes on, so patients with missing data will not be included in upcoming timepoints analysis.

### 2.3 Intervention

**Figure 2:**
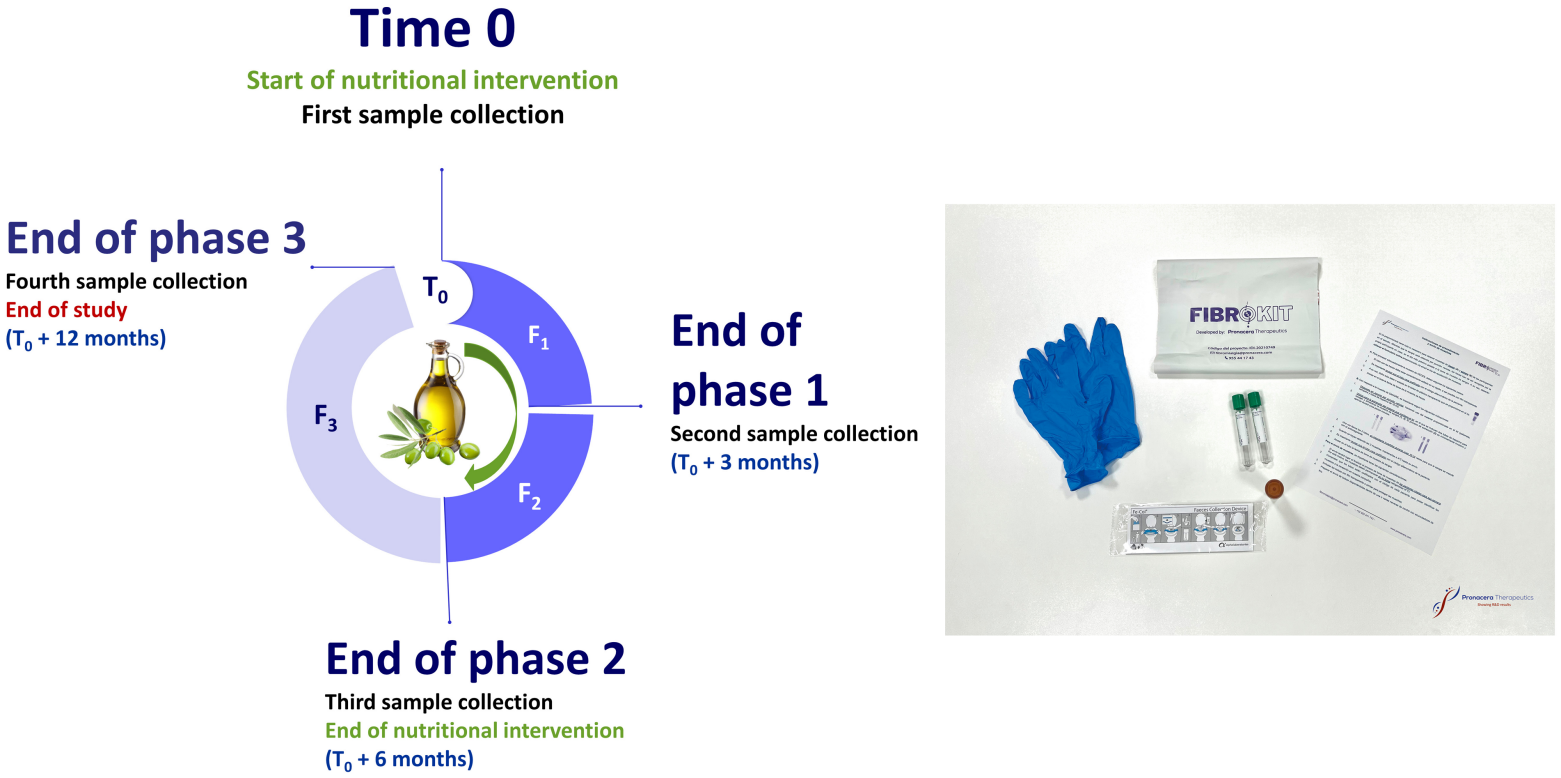
Graphic of the nutritional intervention design and collection kit.

In order to test this new tool as a patient monitoring instrument, the study will investigate the responsiveness of the new panel of markers associated with FM in the assessment and monitoring of treatment. The treatment group will undergo a nutritional intervention based on the Mediterranean diet using EVOO as a supplement, and the placebo group will receive OO. Both groups will be under nutritional control, with regular follow-up calls (twice a month) by their assigned nutritionist. Dietary adherence will be monitored by 24 h dietary recall, as well as a deeper nutritional interview. To avoid excessive withdraw, nutritionist will provided a wide repertoire of foods and cooking tips. The participants will receive a semi-customized list of three-week menus to exchange throughout the intervention (Supplementary material S2). Additionally, they will consume 50 ml of the assigned raw oil, freely distributed through the main meals during the day. This treatment regimen will be administered over a period of 6 months. Figure 2 illustrates the design of the nutritional intervention that will be followed.

### 2.4 Data collection and procedures

#### 2.4.1 Health questionnaires

Participants will complete the Spanish version of the SF-36 health questionnaire following the methodology described by Vilagut et al. (31) in every sample collection, for a total of four data points. This questionnaire aims to assess the participants’ physical and psychological well-being. It consists of 36 questions, referred to therein as items, and covers eight specific scales: physical functioning (PF), role limitations due to physical health (PH), bodily pain (BP), general health perceptions (GH), vitality (V), social functioning (SF), role limitations due to emotional problems (EP) and mental health (MH). Additionally, it includes an item that evaluates participants’ overall health compared to the preceding year. A higher score indicates a better health status. FM patients will complete the fibromyalgia impact questionnaire (FIQ) to assess FM impact on their daily lives. This questionnaire evaluates various aspects, including physical functioning, work performance (missed workdays and work difficulty), depression, anxiety, morning fatigue, pain, stiffness, fatigue and overall well-being during the preceding week. The FIQ consists of 21 items divided into three main domains: function (*n* = 9), overall impact (*n* = 2) and symptoms (*n* = 10). Each item is scored on a scale of zero (no impairment) to 10 (maximum impairment), and the total function score is divided by three. The sum of the scores for overall impact remains unchanged, while the sum of the scores for symptoms is divided by two. The total score of the questionnaire is then calculated by adding these three scores, with the maximum possible total score being 100.

(1) $$FIQ=\frac{\Sigma \mathrm{Function}}{3}+\Sigma \mathrm{Overall Impact}+\frac{\Sigma \mathrm{Symptons}}{2}$$

Questionnaire’s data will be analyzed to search for mean differences. This analysis will help us to strengthen and demonstrate how FM syndrome impact in the quality of life of the patients. Besides, the scores will be used to analyze potential correlations between them and the biomarkers found.

#### 2.4.2 Plasma proteome analysis

Whole blood samples from participants will be collected through venipuncture performed by a healthcare professional. The samples will be collected in two tubes containing heparin-lithium as an anticoagulant. Moreover, to minimize circadian fluctuations of the markers, the blood samples will be taken at the same time of day and participants will have fasted beforehand. While one of the tubes will be used for oxidative stress and mitochondrial metabolism analysis, the other one will be transferred to a 15 ml Falcon tube and then centrifuged at 2,300 RCF for 10 min at 4°C. This process will isolate the plasma, the sample to be used in the proteome analysis that will be aliquoted and stored at −80°C until batch analysis (maximum storing: 5 months). To analyze the plasma proteome, liquid nano-chromatography coupled to the tandem mass spectrometry (nLC-MS/MS) proteomic method will be applied. This analysis will utilize the EvoSEP chromatograph,^1^ which is one of the highest resolution and processing chromatographs available. It is a unique instrument in Spain and is accessible through the services of the BioGUNE Cooperative Research Center in Biosciences located in Biscay, Spain. The processed data will be analyzed with the search engines MASCOT (MatrixScience) and/or PEAKS (Bioinformatics Solutions Inc). Label-free quantification differential proteomics will be performed using PEAKS (Bioinformatics Solutions Inc) and/or MaxQuant software. Quality control of samples will be applied at each step through protein-suitable techniques such as Bradford quantification, electrophoresis and/or Ponceau staining.

#### 2.4.3 Oxidative stress analysis

Oxidative stress levels will be evaluated at the beginning (T_0_) and end (T_F_) of the nutritional intervention by measuring the TAC in whole blood. This will be done using the eBQC device (Bioquochem, Asturias, Spain).^2^ A 100 μL heparin-lithium anticoagulated blood sample will be applied to a test strip, which will then be used to evaluate the global antioxidant status by measuring Q1, Q2 and QT twice. Q1 represents the antioxidant capacity of compounds exhibiting the highest rate of free radical scavenging. These antioxidants are characterized by their rapid action and are the first to undergo oxidation. Notable examples of these fast-acting antioxidants include uric acid, ascorbic acid (vitamin C), GSH, vitamin E, CoQ10 and carotenoids (32, 33). Q2 refers to the antioxidant capacity of compounds that scavenge free radicals at a slower rate. These are known as slow-acting antioxidants. Some examples are polyphenols, alpha-lipoic acid, resveratrol and astaxanthin (32, 33). To quantify the overall antioxidant capacity, the device calculates QT, which results from the combination of contributions from both Q1 and Q2. The eBQC device utilizes electrochemistry to obtain the results. By applying variable voltage, the sample is oxidized to activate the antioxidants. As a result of this activation, electrons are released and then detected to determine the antioxidant capacity (33).

#### 2.4.4 Mitochondrial metabolism analysis

**Figure 3:**
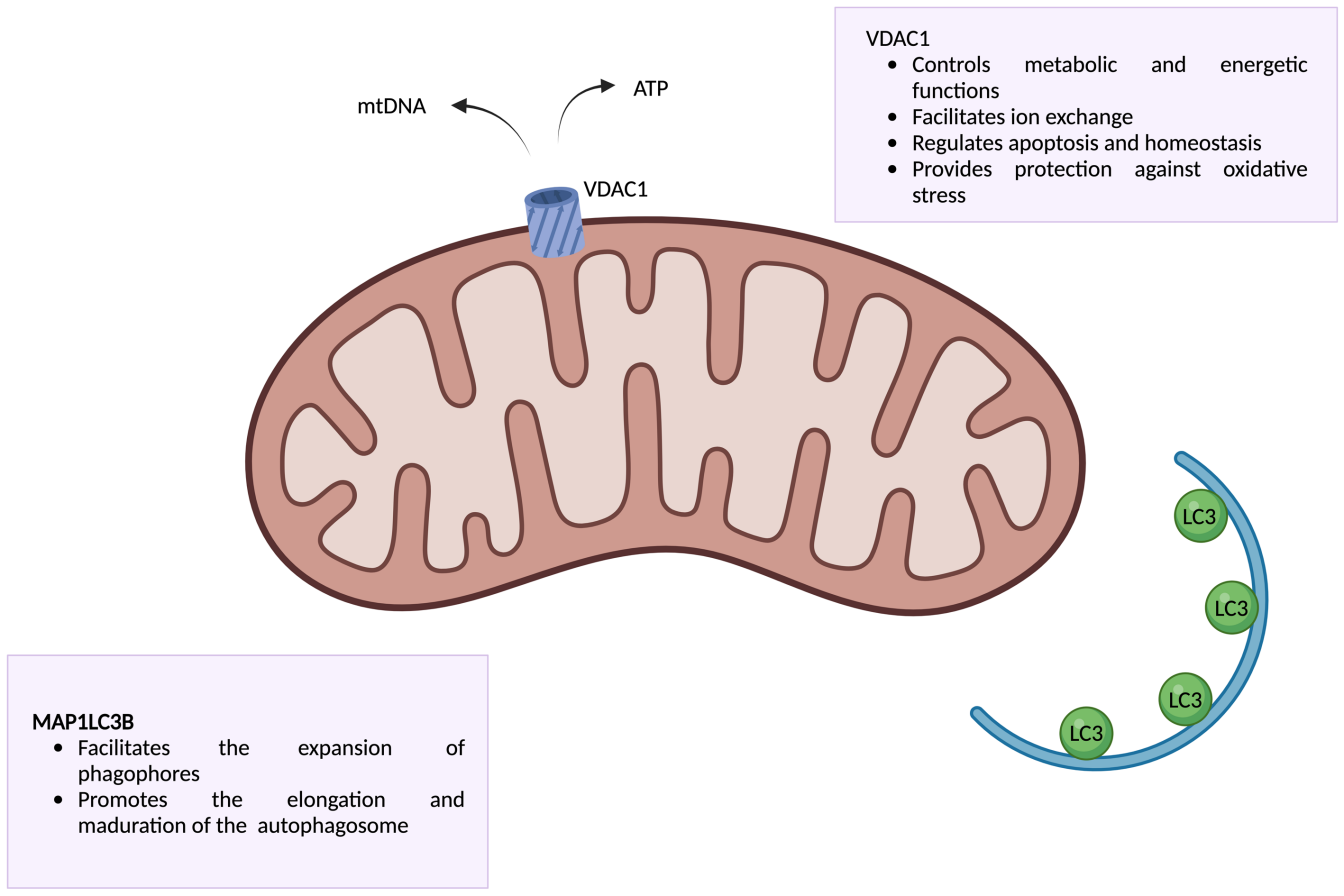
Representation of VDAC and MAP1LC3B proteins along with their functions. Information obtained from Camara et al. (34) and Lee and Lee (35). Created with www.biorender.com.

PBMC will be isolated by a protocol based on ammonium chloride from whole heparin-lithium anticoagulated blood. The samples will be mixed with this solution and then exposed to agitation and centrifugation at 600 RCF for 10 min at 4°C. The resulting cell pellet will be stored at −80°C. Protein extracts will be obtained from PBMC using a protein extraction buffer in white blood cells, containing sucrose 0.32 M, Tris-HCl 0.01 M, magnesium chloride 0.005 M, 1% of Triton-X and protease inhibitors. The separation of these extracts will be carried out using acrylamide gel electrophoresis (SDS-PAGE). The western-blot technique will be used to analyze the abundance of two proteins associated with mitochondrial metabolism: VDAC, a cation channel located in mitochondrial mass in directly linked to mitochondrial mass, and MAP1LC3B (Figure 3), an autophagosome-related protein used to assess autophagy. Additionally, vinculin will be used as a loading control throughout this procedure. To determine mitochondrial ratio, normalized levels of VDAC and MAP1LC3B will be indexed in a numerical algorithm designed with this purpose.

#### 2.4.5 Microbiota and intestinal permeability analysis

Participants will collect fecal samples using the DANASTOOL sample collection kit (Danagen; Barcelona, Spain). They will use a Fe-Col^®^ Faecal sample collection paper (Alpha Laboratories; Hampshire, United Kingdom) as a stool-catch platform. The participants will scoop a small portion of stool into the provided stool tube, which contains a liquid DNA/RNA stabilization solution. Following collection, all samples will be stored in accordance with established quality standards. Since the DANASTOOL sample collection kit has a DNA/RNA stabilization solution, samples were stored at room temperature until batch analysis. DNA extraction from fecal samples will be carried out using the DANAGENE MICROBIOME FECAL DNA KIT (Danagen; Barcelona, Spain). Briefly, 1 ml of homogenized sample was transferred to a tube containing beads to ease sample disruption. Then, samples were incubated at 70°C during 10 min. After incubation step, a thorough vortexing and centrifugation steps were applied. The upper phase was then transferred to a filtering column to extract DNA by centrifugation. The extracted DNA will then be used to analyze the composition of the gut microbiota. This analysis will involve amplicon metagenomics sequencing, in which amplicons of V3–V4 regions from the 16S rDNA will be sequenced using the Illumina MiSeq platform at HelixBioS (Madrid, Spain) facilities. A tagging and barcoding process suitable for Illumina technology will be conducted, according to manufacturer instruction. Each sample will undergo individual quantification, pooling, washing and titration using qPCR. The sequencing will be performed on the MiSeq platform with a fragment size ranging from 2 × 250 to 2 × 300, with the aim of generating 150,000 to 200,000 reads per sample. We will set the minimal number of reads per sample at 50,000 as quantity threshold, and Phred Score at Q30 as quality threshold. Samples with an average trimmed read length below 280 bp will be discarded. All these quality control procedures will be applied through MultiQC software (36) and sequencing results will be analyzed with USEARCH V11.1. Briefly, the reads will be cleaned using the UCHIME algorithm to eliminate “singletons” sequences (those that only had one copy in the total sequences), chimera sequences and possible artifacts. ZOTUs clustering will be carried out using the UNOISE algorithm at 99% identity (similarity). The sequences will be then aligned against the taxonomic database developed for the study of the intestinal microbiota, HUMAN GUT 16S (HelixBioS), with an alignment cut-off point of 99% (identity) using the algorithm USE-LOCAL. The results will be finally processed with phyloseq (37) and DESeq2 (38).

#### 2.4.6 Statistical analysis

The statistical analysis will be performed in RStudio, using R v3.6.2 base packages. First, data distribution will be assessed by using the Kolmogorov–Smirnov test, which evaluates the null hypothesis that data correspond to normal distribution. In that case, in analyses of CONTROL vs. FM groups T_0_, parametric tests such as the unpaired student’s *t*-test and the Pearson correlation test will be used to investigate mean differences and correlation between quantitative variables, respectively. If data do not display normal distribution, the Mann–Whitney *U* test and the Spearman correlation non-parametric test will be applied for this purpose. Additionally, for comparison among groups when depicting nutritional intervention influence with treatment vs. placebo, pre-post analysis through analysis of covariance (ANCOVA) with multiple comparison correction will be used, since it has been shown to be the most powerful analysis for this experimental design (39). In all cases, the significance level (*α*) will be established at 0.05 so that null-hypothesis will be rejected when the *p*-value is <0.05. Tables will be used as preferred format to show descriptive data, such as demographic characteristics, stacked bar charts and/or pie charts will be used to represent distributions, bar charts and/or box and whiskers plots will be applied when showing differences in mean among groups and, finally, violin plots, volcano plots and additional statistical representations will be the format to show distribution and significance in the values found in each group.

## 3 Discussion

FM is a chronic condition that is characterized by pain and for which there is still no precise molecular diagnosis in the healthcare community. Therefore, the main objective of FIBROKIT is to develop a panel of specific biomarkers that can facilitate precise diagnosis and monitoring of the disease. This research will enhance our understanding of the role of oxidative stress, mitochondrial metabolism, intestinal microbiota and inflammation in the pathophysiology of FM. This study introduces a novel approach by designing a comprehensive panel of specific biomarkers for the disease which has not been previously documented. Furthermore, the inclusion of 250 patients represents a significantly larger sample size compared to similar studies, which will help to find relevant results with a strong statistical significance and power. The main goal and greatest challenge of FIBROKIT is the transfer from omics platforms to a group of proteins and microorganisms to be measured by an easy and reproducible wet-lab technique.

Previous studies conducted by this research group have demonstrated an increase in oxidative stress and the presence of mitochondrial dysfunction in FM (40). Furthermore, several studies (10, 41) have reported an increase in the TAC among FM patients, indicating a higher level of oxidative stress in this population. In this context, it is expected that FIBROKIT will find mitochondrial and oxidative markers among its candidates for biomarker discovery. Organelles as well as macromolecules have been widely studied in FM patients. Thus, studies conducted by Ramírez-Tejero et al. (14) and Wåhlén et al. (15) have emphasized the involvement of proteins associated with blood coagulation, immune response and extracellular matrix-receptor interactions, highlighting the role of these pathways in the pathophysiology of the disease and, at the same time, reinforcing the role of proteins as powerful biomarkers in FM. All these responses have been frequently linked to FM, being related with pain, joint stiffness, low-grade chronic inflammation and a severe fatigue (14). Consequently, proteins related with these pathways will serve, not only for diagnostic, but also for personalized treatment for FM patients. In this sense, FIBROKIT will likely involve plasma proteins linked to these pathways, closely related to the main FM symptoms such as pain and fatigue. Focusing on the intestinal microbiota hypothesis, a study (25) has shown reduced bacterial diversity in FM patients, specifically a decrease in *Bifidobacterium* and *Eubacterium* genera. This study also showed disrupted levels of glutamate and serine in FM patients, indicating potential alterations in neurotransmitter metabolism. However, the study conducted by Albayrak et al. (42) revealed significant changes in the patient group, with a statistically significant increase in *Bacteroidia* and *Bifidobacterium*, as well as a decrease in *Firmicutes*. Additionally, this study reported non-significant differences in the abundance of *Enterobacter*, *Streptococcus*, and *Lactobacillus*. A systematic review (43) indicated that the differences in microbiota were found to be associated with both the diagnosis and symptoms of FM. Notably, *Bacteroides* spp. exhibited a positive correlation with the FIQ total symptom score and 2016 ACR diagnostic criteria. Furthermore, there was an increased abundance of *Parabacteroides merdae*, and although not statistically significant, a trend of increased *Akkermansia muciniphila* was also observed. In addition, a separate study has shown that following the Khorasan wheat diet leads to improvements in various FM symptoms, accompanied by alterations in certain gut bacteria (44). This investigation reported positive correlations among *Actinobacteria*, *Verrucomicrobiae*, *Saccharibacteria*, *Bacteroidales* and specific questionnaire scores related to FM symptoms. These findings serve as a basis for the current study, which is expected to obtain results in accordance with those described.

The main study limitation is the marked sex bias. However, this is a predefined bias based on the preponderance of females among FM sufferers. Other study limitations are related to participants’ performance. For instance, self-questionnaires are widely used for nutritional follow-up and health assessment, but it is difficult to determine whether participants are being honest. Additionally, participants’ adherence to the nutritional intervention will be a significant obstacle, as a 1 year study requires significant commitment. Finally, as a multicenter study, coordination of logistical and clinical appointments is presumably a difficult task, and special attention will be required over the course of the project.

In summary, FIBROKIT will be the first study in FM patients to enroll more than 200 patients for biomarker discovery research. At the end of the project, it will be assessed whether plasma and feces may be considered suitable samples in which to look for a differential signature between FM patients and healthy controls. Furthermore, biomarkers’ identification will shed light on molecular diagnosis of FM, as well as will provide potential useful therapeutic targets to deepen in the field of personalized medicine for these patients. Whether theses biomarkers will be applied in clinical practice will need further research and clinical trials with drugs and non-pharmacological therapies.

## Ethics statement

This research, conducted with human participants, underwent a thorough review and received approval from the Ethical Committee for Research with Medications (CEIM) at the Quirónsalud-Catalunya Hospital Group. All participants provided written informed consent prior to their involvement in the study.

## Author contributions

LA: Formal analysis, Writing – original draft. ED-G: Investigation, Methodology, Writing – review & editing. JR-T: Conceptualization, Funding acquisition, Supervision, Writing – review & editing. AM-L: Conceptualization, Funding acquisition, Project administration, Writing – review & editing. DC: Conceptualization, Funding acquisition, Project administration, Writing – review & editing.

## Footnotes

^1^https://www.evosep.com

^2^https://bioquochem.com
