# Corrigendum: Study protocol for FIBROKIT: a new tool for fibromyalgia diagnosis and patient follow-up

**DOI:** 10.3389/fneur.2024.1375764

**Published:** 2024-02-09

**Authors:** Laura Lucena del Amo, Elena Durán-González, Jorge A. Ramírez-Tejero, Antonio Martínez-Lara, David Cotán

**Affiliations:** Pronacera Therapeutics S.L., Seville, Spain

**Keywords:** fibromyalgia, gut microbiota, oxidative stress, proteomics, mitochondria, metagenomics

In the published article, there was an error. The number of pain points was incorrectly reported.

A correction has been made to Introduction, paragraph number 1. This sentence previously stated:

“Traditionally, exploration of tender points was also used for diagnosis, establishing the threshold at a minimum of 11 out of 11 pain points, although this diagnostic procedure is becoming outdated.”

The corrected sentence appears below:

“Traditionally, exploration of tender points was also used for diagnosis, establishing the threshold at a minimum of 11 out of 18 pain points, although this diagnostic procedure is becoming outdated.”

In the published article, there was an error in Figure 2 as published. A typographical error “Forth sample collection” instead of “Fourth sample collection” was erroneously written. The corrected Figure 2 appears below.

**Figure 2:**
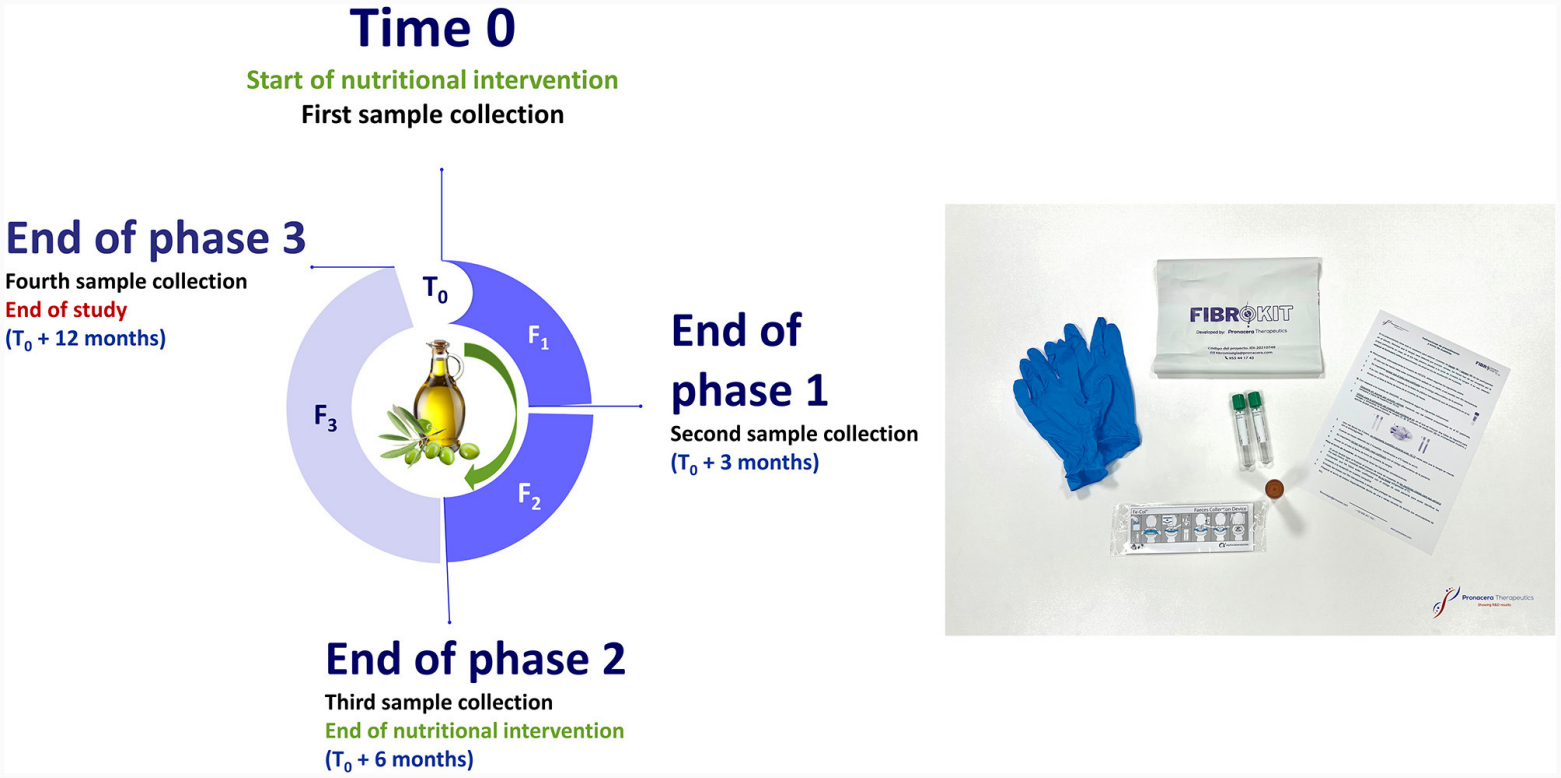
Graphic of the nutritional intervention design and collection kit.

The authors apologize for this error and state that this does not change the scientific conclusions of the article in any way. The original article has been updated.

