# Supplementary material for: Study protocol for FIBROKIT: a new tool for fibromyalgia diagnosis and patient follow-up
Source: Front Neurol. 2023 Nov 21;14:1286539. doi: 10.3389/fneur.2023.1286539 (PMC10710143; doi:10.3389/fneur.2023.1286539)

Pronacera Therapeutics
Showing R&D results

Grupo CINUSA
CENTROS DE INVESTIGACIÓN EN NUTRICIÓN Y SALUD

VALIDATION OF BIOMARKERS RELATED TO FIBROMYALGIA AND THEIR USEFULNESS IN THE DIAGNOSIS AND FOLLOW-UP OF PATIENTS

Dietary and washout guidelines for participants prior to the start of the intervention:

- It is recommended to eat at least 3 pieces of fruit daily and whole, preferably seasonal and proximity. Avoid juices
- Vegetables should be eaten for lunch and dinner, you can always change one vegetable for another one or you can modify the type of preparation (it is similar to taking a cream as a raw vegetable, for example).
- Cereals, bread, flours and pasta, preferably whole grain or whole grain.
- You can accompany meals with bread.
- Select natural nuts, e.g. not fried or salted. It is recommended to take several servings a week.
- To drink, choose water preferably. Drink between 4-6 glasses a day.
- For dessert you can always have fruit, yogurt, an ounce of chocolate or tea.
- Natural yogurts (preferably unsweetened and unsweetened) are recommended.
- Fish can be swapped for each other, as can meat.
- Rice, potato, pasta, and quinoa can be swapped for each other.
- Vegetables (greens) can be swapped for each other.
- Salads on the menu: you can combine lettuce, lamb's lettuce, arugula, endives, tomatoes, cucumbers, peppers, carrots, peppers, spring onions, beetroot, kale, green beans...
- It is recommended to consume legumes at least twice a week, the same time can be repeated. Avoid fatty preparations (such as adding bacon or chorizo) and they can also be eaten in salads.
- The way of cooking or preparation or presentation suggestion, are just a suggestion, you can change it for another that is to your liking.
- In the mid-mornings and snacks you can combine several options, if you are not hungry you do not need to do it.
- **Important:** The washing period is 2 weeks. You can choose to follow any of the 3 options presented here during that period. Once the washing period is over, you will be provided with the corresponding oil (Type A or Type B) and you must consume it as indicated in the 3 menus listed below.
- **If you have any questions or suggestions regarding the menus, please contact any of the following emails:** info@grupocinusa.es / fibromialgia@pronacera.com

Pronacera Therapeutics
Showing R&D results

Grupo CINUSA
CENTROS DE INVESTIGACIÓN EN NUTRICIÓN Y SALUD

Week 1

	Monday	Tuesday	Wednesday	Thursday	Friday	Saturday	Sunday
Breakfast	To drink: Infusion/Milk/Vegetable drink with no added sugar<br>You can accompany it with:<br>Option 1: Fruit<br>Option 2: Toast **with olive oil** and/or tomato<br>Option 3: Avocado + **Olive Oil Toast**<br>Option 4: Toast with fresh cheese and tomato + with **olive oil**<br>Option 5: Cereal flakes<br>Option 6: Plain yoghurt with cereal flakes<br>You can optionally add fruit to options 2,3,4, 5 and 6						
Mid-morning	Option 1: Fruit<br>Option 2: A handful of natural nuts<br>Option 3: Toast with olive oil and/or tomato and/or fresh cheese and/or serrano ham / and/or turkey + with olive oil<br>Option 4: Plain yogurt						
Food	Salad + **olive oil**<br><br>Lentils cooked with vegetables	Baked aubergine stuffed with vegetables, minced meat, natural crushed tomato and mozzarella	Salmon papillote with roasted potato + **olive oil**<br><br>Roasted zucchini and butternut squash	Salad + **olive oil**<br><br>Chickpeas with vegetables	Turkey tenderloin with pepper and roasted apple<br><br>Vegetable stew	Pasta with vegetables, natural crushed tomato and prawns + with **olive oil**	Salad + **olive oil**<br><br>Vegetable paella with chicken
Snack	Option 1: Fruit<br>Option 2: A handful of natural nuts<br>Option 3: Toast with olive oil and/or tomato and/or fresh cheese and/or serrano ham / and/or turkey + with olive oil<br>Option 4: Plain yogurt<br>Option 5: Plain yogurt with pure seeds/fruit/cocoa						
Dinner	Grilled vegetables with quinoa and grilled egg	Grilled sea bream with spring onion<br><br>Mushrooms + **olive oil**	Cream of zucchini + **olive oil**<br><br>Omelette	Roasted pepper salad with spring onion and tuna belly + **olive oil**	Grilled cuttlefish with parsley<br><br>Salad + **olive oil**	Potato omelette<br><br>Green beans with carrot + **olive oil** and apple cider vinegar	Grilled wild asparagus + **olive oil**<br><br>Grilled sole

Pronacera Therapeutics
Showing R&D results

Grupo CINUSA
CENTROS DE INVESTIGACIÓN EN NUTRICIÓN Y SALUD

Week 2

	Monday	Tuesday	Wednesday	Thursday	Friday	Saturday	Sunday
Breakfast	To drink: Infusion/Milk/Vegetable drink with no added sugar<br>You can accompany it with:<br>Option 1: Fruit<br>Option 2: Toast **with olive oil** and/or tomato<br>Option 3: Avocado + **Olive Oil Toast**<br>Option 4: Toast with fresh cheese and tomato + with **olive oil**<br>Option 5: Cereal flakes<br>Option 6: Plain yoghurt with cereal flakes<br>You can optionally add fruit to options 2,3,4, 5 and 6						
Mid-morning	Option 1: Fruit<br>Option 2: A handful of natural nuts<br>Option 3: Toast with olive oil and/or tomato and/or fresh cheese and/or serrano ham / and/or turkey + with olive oil<br>Option 4: Plain yogurt						
Food	Cod with natural crushed tomato, spring onion, potato and salad + **olive oil**	Roast Chicken with Vegetables<br><br>Rice garnish	Salad + **olive oil**<br><br>Lentils cooked with vegetables	Grilled tuna with sesame seeds<br><br>Salad with avocado + **olive oil**	Salad + **olive oil**<br><br>White Beans Cooked with Vegetables	Salad + **olive oil**<br><br>Seafood noodles	Pumpkin cream + **olive oil**<br><br>Baked pork tenderloin
Snack	Option 1: Fruit<br>Option 2: A handful of natural nuts<br>Option 3: Toast with olive oil and/or tomato and/or fresh cheese and/or serrano ham / and/or turkey + with olive oil<br>Option 4: Plain yogurt<br>Option 5: Plain yogurt with pure seeds/fruit/cocoa						
Dinner	Scrambled eggs with mushrooms and spring onion	Homemade soup with noodles<br><br>Grilled rooster with spices	Sautéed spinach with potatoes and serrano ham cubes	Grilled zucchini + **olive oil**<br><br>Grilled chicken	Carrot cream + **olive oil**<br><br>Baked sea bass	Homemade Chicken Burger<br><br>Grilled vegetables	Omelette<br><br>Homemade vegetable ratatouille

Week 3

<table>
<tr><th></th><th>Monday</th><th>Tuesday</th><th>Wednesday</th><th>Thursday</th><th>Friday</th><th>Saturday</th><th>Sunday</th></tr>
<tr><td>Breakfast</td><td colspan="7">To drink: Infusion/Milk/Vegetable drink with no added sugar
You can accompany it with:
Option 1: Fruit
Option 2: Toast with olive oil and/or tomato
Option 3: Avocado + Olive Oil Toast
Option 4: Toast with fresh cheese and tomato + with olive oil
Option 5: Cereal flakes
Option 6: Plain yoghurt with cereal flakes
You can optionally add fruit to options 2,3,4, 5 and 6</td></tr>
<tr><td>Mid-morning</td><td colspan="7">Option 1: Fruit
Option 2: A handful of natural nuts
Option 3: Toast with olive oil and/or tomato and/or fresh cheese and/or serrano ham / and/or turkey + with olive oil
Option 4: Plain yogurt</td></tr>
<tr><td>Food</td><td>Salad + olive oil

Lentils cooked with vegetables</td><td>Beef stew with potato, peas and vegetables</td><td>Baked zucchini stuffed with quinoa, vegetables and tuna, mozzarella</td><td>Salad + olive oil

Cooked</td><td>Rice with turmeric, vegetables (carrot, zucchini...), chicken</td><td>Baked hake with clams and roasted potato

Roasted vegetables + olive oil</td><td>Pasta with vegetables (wild asparagus, spring onion...) and salmon + olive oil</td></tr>
<tr><td>Snack</td><td colspan="7">Option 1: Fruit
Option 2: A handful of natural nuts
Option 3: Toast with olive oil and/or tomato and/or fresh cheese and/or serrano ham / and/or turkey + with olive oil
Option 4: Plain yogurt
Option 5: Plain yogurt with pure seeds/fruit/cocoa</td></tr>
<tr><td>Dinner</td><td>Vegetable stew

Grilled chicken</td><td>French omelette with prawns

Cream of mushroom soup + olive oil</td><td>Artichokes + olive oil

Grilled chicken</td><td>Potato omelette

Pumpkin cream + olive oil</td><td>Green beans with tomato

Turbot</td><td>Spinach with egg

Vegetable soup</td><td>Grilled turkey tenderloin with pepper

Sautéed Swiss chard</td></tr>
</table>

GENERAL AND ORGANIZATIONAL INFORMATION:

You will receive **weekly rotating menu planning** so that you can have a **balanced, complete and nutritious diet** with the support of registered Dietitians-Nutritionists.

In addition, during the 6 months of nutritional intervention of the study, you will receive the daily dose of olive oil that you must include in your diet every day, **raw** (without being able to be used in culinary preparations). These rigid plastic sachets have a capacity of 50 ml, which is approximately equivalent to 5 tablespoons.

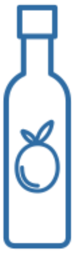

In order to **comply with the daily indications** of the oil intake proposed in the study, here are some **examples**:

1) Toast of bread with oil:

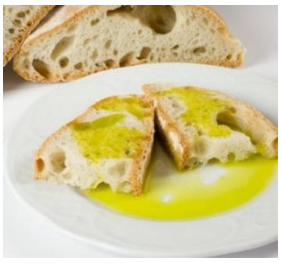
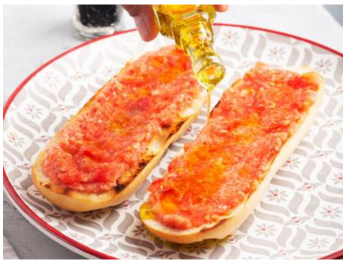
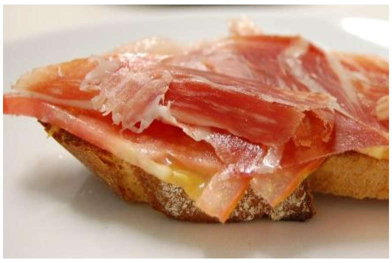
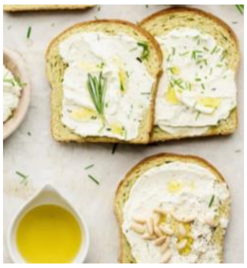
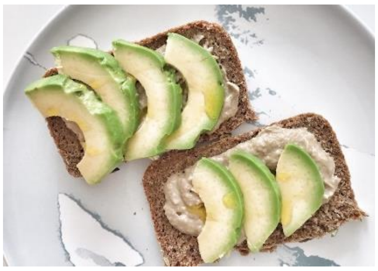
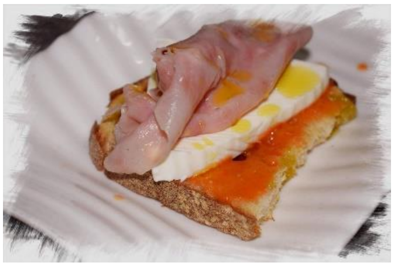

2) Splash of oil as a dressing in salads:

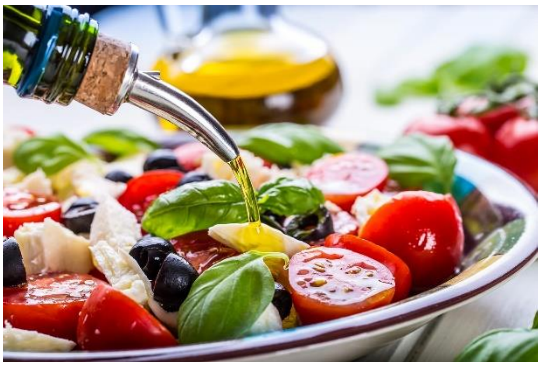

3) Splash of oil as a presentation in creams or salmorejo / gazpacho:

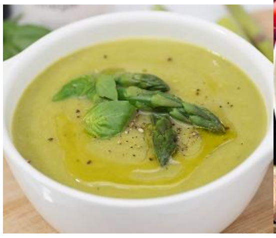
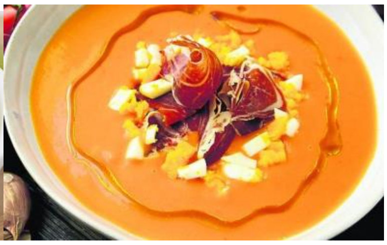
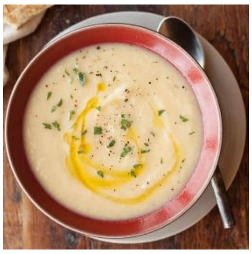

4) Splash of oil on cooked vegetables (steamed/boiled):

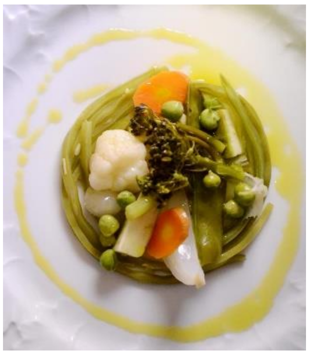
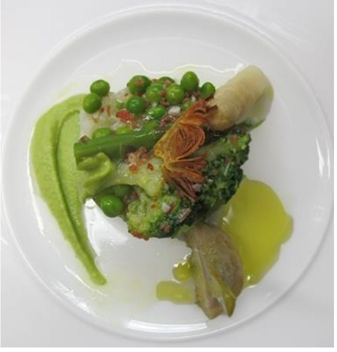

5) Splash of oil as a presentation in boiled or roasted potatoes:

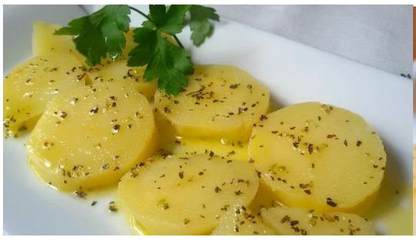
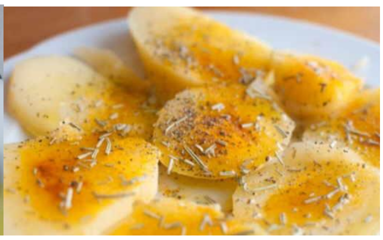

Supplement: Supplementary file 1 [file Data_Sheet_1.PDF]
