# Supplementary material for: Study protocol for FIBROKIT: a new tool for fibromyalgia diagnosis and patient follow-up
Source: Front Neurol. 2023 Nov 21;14:1286539. doi: 10.3389/fneur.2023.1286539 (PMC10710143; doi:10.3389/fneur.2023.1286539)
Supplement: Supplementary file 2 [file Data_Sheet_2.PDF]

INFORMED CONSENT FOR RESEARCH STUDY

1. PRINCIPAL INVESTIGATOR

Name: Dr. José Horcajadas
Centre: Pronacera Therapeutics S.L.
Position: Scientific Advisor
Contact: jose.horcajadas@gmail.com

2. RESEARCH SUMMARY

**Project Title**	CLINICAL TRIAL FOR THE SEARCH FOR BIOMARKERS RELATED TO FIBROMYALGIA AND THEIR USEFULNESS IN THE DIAGNOSIS AND FOLLOW-UP OF PATIENTS DURING NUTRITIONAL INTERVENTION WITH OLIVE OIL (Code: 20210749)*
**Promoter**	Pronacera Therapeutics S.L.
**Financer**	Centre for the Development of Industrial Technology (CDTI)
**Place(s)**	Pronacera Therapeutics S.L.: Parque Empresarial Arte Sacro de Sevilla (PEASS), Avenida de la Ingeniería, 9 - Nave 34, C.P. 41015 (Sevilla)<br>Centros de Investigación en Nutrición y Salud S.L., CINUSA: Paseo de la Habana, 43, C.P. 28036 (Madrid)<br>BioGUNE Cooperative Research Centre: Bizkaia Science and Technology Park, Building 801A, C.P. 48160 Derio (Vizcaya).<br>HelixBios S.L.: Parque Científico de Madrid, Calle Faraday, 7, C.P. 28049 (Madrid)<br>Medical centres for sampling: SYNLAB (Seville, Badajoz, Madrid and Oviedo) and Laboratory of Dr. Esther Cobo López (Jaén)
**Purpose**	Selection of a Biomarker Panel to Support the Diagnosis and Monitoring of Fibromyalgia Patients

**Study approved by the Research Ethics Committee in accordance with the Biomedical Research Law of 14/2007, of 3 July.*

1. INFORMED CONSENT

I____
______

☐ I have read the information sheet and Appendix 1 that has been given to me about the study.
☐ I've been able to ask questions about the study.
☐ I have received enough information about the study.
☐ I have spoken with
____

☐ I understand that my participation is voluntary.
☐ I understand that I can withdraw from the study:
-Whenever.
- No having to explain.
- This does not affect my medical care.

☐ I consent to the storage and use of biological samples (surplus or not, it can be specified depending on the case) and associated data for future research under the conditions explained in the information sheet.

☐ I consent to be contacted in the event that I need further information or additional biological samples.

I will receive a signed and dated copy of this information and informed consent sheet.

I freely agree to participate.

In ...., to .... of....................... of 20.....

Participant's signature: Signature of the healthcare professional/researcher:

Fdo.: Fdo.:

PATIENT INFORMATION SHEET FOR RESEARCH STUDY

1. PRINCIPAL INVESTIGATOR

Name: Dr. José Horcajadas
Position: Scientific Advisor
Centre: Pronacera Therapeutics S.L.
Contact: jose.horcajadas@gmail.com

2. RESEARCH SUMMARY

**Project Title**	CLINICAL TRIAL FOR THE SEARCH FOR BIOMARKERS RELATED TO FIBROMYALGIA AND THEIR USEFULNESS IN THE DIAGNOSIS AND FOLLOW-UP OF PATIENTS DURING NUTRITIONAL INTERVENTION WITH OLIVE OIL (Code: 20210749)*
**Promoter**	Pronacera Therapeutics S.L.
**Financer**	Centre for the Development of Industrial Technology (CDTI)
**Place(s)**	Pronacera Therapeutics S.L.: Parque Empresarial Arte Sacro de Sevilla (PEASS), Avenida de la Ingeniería, 9 - Nave 34, C.P. 41015 (Sevilla)<br>Centros de Investigación en Nutrición y Salud S.L., CINUSA: Paseo de la Habana, 43, C.P. 28036 (Madrid)<br>BioGUNE Cooperative Research Centre: Bizkaia Science and Technology Park, Building 801A, C.P. 48160 Derio (Vizcaya).<br>HelixBios S.L.: Parque Científico de Madrid, Calle Faraday, 7, C.P. 28049 (Madrid)<br>Medical centres for sampling: SYNLAB (Seville, Badajoz, Madrid and Oviedo) and Laboratory of Dr. Esther Cobo López (Jaén)
**Purpose**	Selection of a Biomarker Panel to Support the Diagnosis and Monitoring of Fibromyalgia Patients

**Study approved by the Research Ethics Committee in accordance with the Biomedical Research Law of 14/2007, of 3 July.*

3. INTRODUCTION

We are writing to inform you about a research study in which you are invited to participate. The study has been approved by a Research Ethics Committee, in accordance with current legislation. We want you to receive accurate and sufficient information so that you can **decide whether or not to participate in this study**. To do this, please read this fact sheet carefully and we will clarify any doubts you may have. In addition, you can consult with the people you see fit

4. VOLUNTARY PARTICIPATION

The study detailed here aims to **evaluate a set of markers** that can help **diagnose and monitor people with fibromyalgia** who eat a **balanced diet,** supplemented **with Extra Virgin Olive Oil** (EVOO). To do this, we will work on a group of 210 fibromyalgia (FM) patients and 40 healthy volunteers, belonging to a defined population group to which you belong. You should know that **your participation in this study is voluntary and that you can choose NOT to participate**. If you choose to participate, you can change your decision and withdraw consent at any time, without altering your relationship with your doctor or harming your health care.

5. ABOUT THE STUDY

OBJECTIVE

Fibromyalgia (FM) is a **chronic disease** related to many factors and characterized by pain and a wide variety of physical and psychological symptoms. Due to the lack of knowledge about its origin and the diversity of its symptoms, it is difficult to find associated molecular markers, which complicates its diagnosis and treatment. Although the molecular changes are still unclear, many studies have pointed to oxidative stress, inflammation, and imbalance in the ratios of certain gut bacteria as potential biomarkers of FM. Similarly, it has been shown that the intake of foods beneficial to human health (such as EVOO) are able to reduce the level of several of these indicators in people with FM. Thus, the objective of this study is to **identify biomarkers** that can help **diagnose and monitor the** response of **people with fibromyalgia** to the consumption of a **balanced diet,** supplemented **with EVOO.** This information will be extremely useful for support in the diagnosis and follow-up of people with FM, laying the foundations for progress in their research.

DESCRIPTION

It is planned to recruit 210 FM patients and 40 healthy volunteers. Participation in the study will have a total duration of 12 months. During the first 6 years, you agree to maintain a balanced diet, being advised completely free of charge by our qualified and registered nutritionists, which will be **supplemented with EVOO** (50 ml/day) in the case of the

"TREATMENT" group, or with **Olive Oil** (50 ml/day) **in the case of the "PLACEBO" group** . Your assignment to the appropriate group will be done at random, and neither you nor the participating researcher will know what type of oil you will be consuming. Samples will be needed at Time 0 (start), Time 1 (3rd month), Time 2 (6th month) and Time 3 (12th month) from:

1. **Blood:** drawn by the usual means (puncture in a vein) by health professionals. It will be held on an empty stomach, always in the morning. 2 blood samples will be taken and the amount extracted in each analysis will be 20 ml of blood (2 tubes of 10 ml). For most people, needle sticks for blood collection are not a problem. However, occasionally, they can cause bleeding, bruising, discomfort, infection, and/or pain at the blood collection site. You may also feel dizzy.
2. **Faeces**: you will receive at home a self-collection kit with all the necessary utensils to carry out the sampling in healthy and sterile conditions. This sample will be delivered to the medical center on the same days as the blood draw.

The samples will be associated with a code that can only be related to their identity by authorized personnel (researchers participating in the study by the sponsoring company), in the same way as previously explained with the data obtained during the trial. The data derived from the use of these samples will be treated in the same way as the rest of the data obtained during this test (see section on the protection of personal data). Samples and associated data shall be maintained under appropriate security conditions and it is ensured that subjects may not be identified through means considered reasonable by persons other than those authorized. Some additional data or samples may be required. In this case, the responsible investigator will contact you to ask for your cooperation again.

As a result of the procedure, you will be nutritionally advised by a health professional for 6 months, receiving during this time and free of charge the supplement that corresponds to you to consume according to the group that is assigned to you (EVOO/Olive Oil). During this period, you also have the right to know (if you wish) the evolution of parameters studied in this project, such as the state of your gut microbiota or your plasma proteomic profile.

RISKS AND INCONVENIENCES ARISING FROM YOUR PARTICIPATION IN THE STUDY

Since the study is based on a nutritional intervention supplemented with commercial olive oil suitable for human consumption, no type of risk or discomfort is foreseen during it, beyond the one derived from the taking of a blood sample (puncture). By participating in this study, you agree to:

1. **Follow the nutritional recommendations made by the professionals coordinating the study**, notifying them,

whenever possible, of any significant deviation you have decided to take.

2. **It waives any economic, patrimonial or optional right over the results or potential benefits that** may be derived directly or indirectly from the research carried out with the sample that is ceded for research.

POSSIBLE BENEFITS

As a result of nutritional counselling, it is possible that your diet will improve, with the benefits that this entails (weight control, lighter digestion, improved meal regime, etc.). However, you may not get any benefit to your health.

OBLIGATIONS OF THE PARTICIPATING COMPANY(IES)

Pronacera Therapeutics S.L., as well as the collaborating centers in which they will be taken (Synlab, Clinical Analysis Laboratory of Dr. Esther Cobo) and processed (CINUSA; BioGUNE; HelixBios) samples are required to:

1. **Guarantee the participation in the process of duly qualified and collegiate professionals,** always covered under the respective Civil Liability Insurance that allows to cover any type of incident that arises during the study.
2. **Ensure the confidentiality of the data obtained in the study**, which will be stored and duly coded in the custody of Pronacera Therapeutics S.L., solely for research purposes.
3. **Control access to the data obtained in the study**, a privilege that can only be exercised by those belonging to the aforementioned companies.

RIGHTS AND OBLIGATIONS OF THE PARTICIPANT

At all times, you have the right to:

1. **Exercise your rights of access, rectification, cancellation and opposition** to the data collected during the study.[1]
2. **Revoke consent and unilaterally abandon the study without prior notice or justification**.[1]
3. **Request the destruction of all your data and samples.**[1]

1In order to duly exercise any of the rights set out in these first 3 sections, it will be sufficient to transfer this request to the company

(Pronacera Therapeutics S.L.) by any means of communication (ordinary mail: Parque Empresarial Arte Sacro y Afines de Sevilla, Local 34, C.P.41015, Seville; e-mail: info@pronacera.com).

4. To request any type of complementary or additional information to the study, contact the researchers responsible for the study, who will assist you as soon as possible.

PLACE OF ANALYSIS AND STORAGE OF SAMPLES

Peripheral blood and stool samples obtained for this study will be used only for biomedical research. These will be pre-processed and stored for research purposes for 25 years at the facilities of Pronacera Therapeutics (Parque Empresarial Arte Sacro de Sevilla, Local 34, C.P.41015), where they will become part of a collection of biological samples of human origin for biomedical research purposes (as defined in Royal Decree 1716/2011) in anticipation of the need to repeat any additional analysis related to the objectives of the trial. unless you ask us otherwise. During this process, the person responsible for the samples will be the promoter of the trial. The analysis phase will take place in the collaborating companies, depending on the nature of the sample (the blood will be analysed at the BioGUNE Cooperative Research Centre, while the faeces will be evaluated at HelixBios S.L.). Likewise, in order to guarantee their confidentiality without compromising the correct analysis of the samples, they will undergo a coding process, consisting of the assignment of an alphanumeric code to your samples, the link of which will only be recovered in exceptional cases, such as, for example, the request of your own personal data by you.

FUTURE USE OF THE SAMPLES

The samples will be stored in the biological sample collection of the IDI-20210749 project (located in the facilities of Pronacera Therapeutics, at Avenida de la Ingeniería, nº9 - Local 34 - of the Arte Sacro Business Park in Seville), they will not be transferred to third parties, and they will be used in projects favorably reported by a Research Ethics Committee, and related to the line of research in fibromyalgia of the promoter company. In the event that the use or transfer of their samples in a different research is considered, their consent will be requested. You can contact Mr. Jorge Antolín Ramírez Tejero (fibromialgia@pronacera.com; 955 44 17 43) for information on the projects in which your samples have been used.

RIGHT TO REVOKE CONSENT

If you change your mind regarding the donation of the biological samples and the transfer of the data provided, you have the right to request their destruction or anonymization, through the principal investigator of the biological sample collection of the IDI-20210749 project. However, you should be aware that the data obtained in the analyses carried out up to that point may be used for the purposes requested and may be kept in compliance with the corresponding legal obligations.

CONTACT IN CASE OF QUESTIONS

If you have any questions or need more information during your participation, please contact Mr. Jorge Antolín Ramírez Tejero, PhD in Molecular Biology, Technical Director of the company promoting the study and head of the research line, by calling 955 441 743 from Monday to Friday from 9:00 a.m. to 6:00 p.m. or by mail to fibromialgia@pronacera.com. In case of urgency or emergency, go quickly to your usual medical center.

EXPENSES AND FINANCIAL COMPENSATION

Your participation in this study is voluntary and altruistic, so you will NOT receive any financial compensation for it. The sponsor of the study is responsible for managing the financing of the study. To carry out the study, the sponsor of the study has signed a contract with the doctor of the study and center where it is going to be carried out. You will not have to pay for the oil supplied or for specific tests of the study. Your participation in the study will not entail any additional expense to the usual clinical practice and you will be reimbursed for the extraordinary expenses that participation in the study generate.

6. PROTECTION OF PERSONAL DATA

Your identity and all personal data collected will be masked from the moment it is collected, in accordance with current regulations on the protection of personal data (European Regulation 2016/679 on the protection of natural persons with regard to the processing of personal data; LO 3/2018 on the Protection of Personal Data and Guarantee of Digital Rights -Seventeenth Additional Provision, relating to the processing of health data-). Both the promoter and the centre will ensure that the principles set out in both national and European data protection regulations are complied with.

What will my data be used for?

Your data is necessary for the sponsor to continue its line of research into the biological basis of fibromyalgia. Therefore, they will be used as planned in this study, as well as within the related research activities necessary for this research, to:

- better understand the disease studied and the associated health problems,
- Develop diagnostic tests for the disease
- learn from previous studies to plan new studies or improve methods of scientific analysis
- publish the results in scientific journals or use them for educational purposes

7. MANIFEST

Having read and understood the above, I declare that:

1. I understand the study, its associated procedures, the probabilities of its success, the associated risks and the possible complications of the proposed methodology.

2. I am aware of the willingness of research and health personnel to attend to any aspect of the information that has not been sufficiently clear at any time during the study.

3. I have understood the explanations that have been provided to me in clear and simple language, and the doctor and/or research staff who have attended me have allowed me to make all the observations that I have considered appropriate, and has clarified all the doubts raised.

4. I am satisfied with the information received **and I freely provide, and without financial compensation derived from my participation in the study, my agreement for my biological samples** (blood and feces) to be collected and processed at the Center/Clinic:.................................., as well as for these to be sent to the premise of Pronacera Therapeutics S.L. for research.

In ...., to .... of........................ of 20.....

Participant's signature: Signature of the healthcare professional/researcher:

Fdo.: Fdo.:
